# Affective and cognitive behavior in the alpha-galactosidase A deficient mouse model of Fabry disease

**DOI:** 10.1371/journal.pone.0180601

**Published:** 2017-06-29

**Authors:** Lukas Hofmann, Franziska Karl, Claudia Sommer, Nurcan Üçeyler

**Affiliations:** Department of Neurology, University of Würzburg, Würzburg, Germany; Universita degli Studi dell'Insubria, ITALY

## Abstract

Fabry disease is an X-linked inherited lysosomal storage disorder with intracellular accumulation of globotriaosylceramide (Gb3) due to α-galactosidase A (α-Gal A) deficiency. Fabry patients frequently report of anxiety, depression, and impaired cognitive function. We characterized affective and cognitive phenotype of male mice with α-Gal A deficiency (Fabry KO) and compared results with those of age-matched male wildtype (WT) littermates. Young (3 months) and old (≥ 18 months) mice were tested in the naïve state and after i.pl. injection of complete Freund`s adjuvant (CFA) as an inflammatory pain model. We used the elevated plus maze (EPM), the light-dark box (LDB) and the open field test (OF) to investigate anxiety-like behavior. The forced swim test (FST) and Morris water maze (MWM) were applied to assess depressive-like and learning behavior. The EPM test revealed no intergroup difference for anxiety-like behavior in naïve young and old Fabry KO mice compared to WT littermates, except for longer time spent in open arms of the EPM for young WT mice compared to young Fabry KO mice (p<0.05). After CFA injection, young Fabry KO mice showed increased anxiety-like behavior compared to young WT littermates (p<0.05) and naïve young Fabry KO mice (p<0.05) in the EPM as reflected by shorter time spent in EPM open arms. There were no relevant differences in the LDB and the OF test, except for longer time spent in the center zone of the OF by young WT mice compared to young Fabry KO mice (p<0.05). Complementary to this, depression-like and learning behavior were not different between genotypes and age-groups, except for the expectedly lower memory performance in older age-groups compared to young mice. Our results indicate that genetic influences on affective and cognitive symptoms in FD may be of subordinate relevance, drawing attention to potential influences of environmental and epigenetic factors.

## Introduction

Fabry disease (FD) is an X-linked inherited lysosomal storage disorder with intracellular accumulation of globotriaosylceramide (Gb3) due to an α-galactosidase A (α-Gal A) deficiency or a complete loss of enzyme function [1]. Fabry patients mainly develop a peripheral neuropathy of the small fiber type and characteristically suffer from episodic acral burning pain particularly triggered by heat and fever [2]. Fabry-associated pain substantially limits health related quality of life already starting in early childhood [3, 4].

In addition to pain, Fabry patients frequently report of affective symptoms like anxiety, depressed mood, and cognitive impairment such as deficits in memory, attention or psychomotor performance. In contrast to such clinical reports, systematic data on affective symptoms and cognitive functions in FD patients are scarce [5]. While in most studies depressive symptoms were found in 15–60% of the investigated FD patients [4, 6–10], data on cognitive impairment are conflicting and rather point to mild and non-progressive alterations [4, 6, 7]. However, interpretation of these results remains difficult particularly due to the diversity of methods used. Also, appropriate tools reflecting the distinct neuropsychological phenotype of FD patients observed in clinical practice are lacking [5].

One key question is, if the affective and cognitive symptoms in FD are part of disease pathophysiology or a reactive process developing in the course of a life-threatening, chronic, and often painful disorder. Assessing causative treatment responses would be one possibility to answer this question. However, current clinical studies only allow limited insights, since neuropsychological changes under treatment, e.g. enzyme replacement therapy, are mostly subordinate end-points that may secondarily be influenced by treatment-associated improvement in organ function. Investigating affective and cognitive behavior in mouse models of FD [11–13] might thus be a useful alternative. These mice have been assessed mainly for organ pathology and pain [14–17], however, no data are available on affective and cognitive behavior so far.

Using the α-GAL A deficient mouse model (Fabry KO) [18] we set out to comprehensively characterize anxiety-, depression-, and learning-behavior in Fabry KO mice compared to their wild-type littermates (WT). We assessed mice naïve and after injection of complete Freund`s adjuvant (CFA) as a model of inflammatory pain also inducing anxiety [19, 20], to answer the question if the Fabry genotype itself is associated with neuropsychological and emotional deficits.

## Methods

### Ethic statement

Our experiments were approved by the Bavarian State authorities (Regierung von Unterfranken, # 54/12). Animal use and care were in accordance with the institutional guidelines. Mice were housed in the animal facilities of the Department of Neurology, University of Würzburg; they were fed commercially prepared complete diet (standard chow) and had food and water access ad libitum.

### Mice and treatment

**Fig 1:**
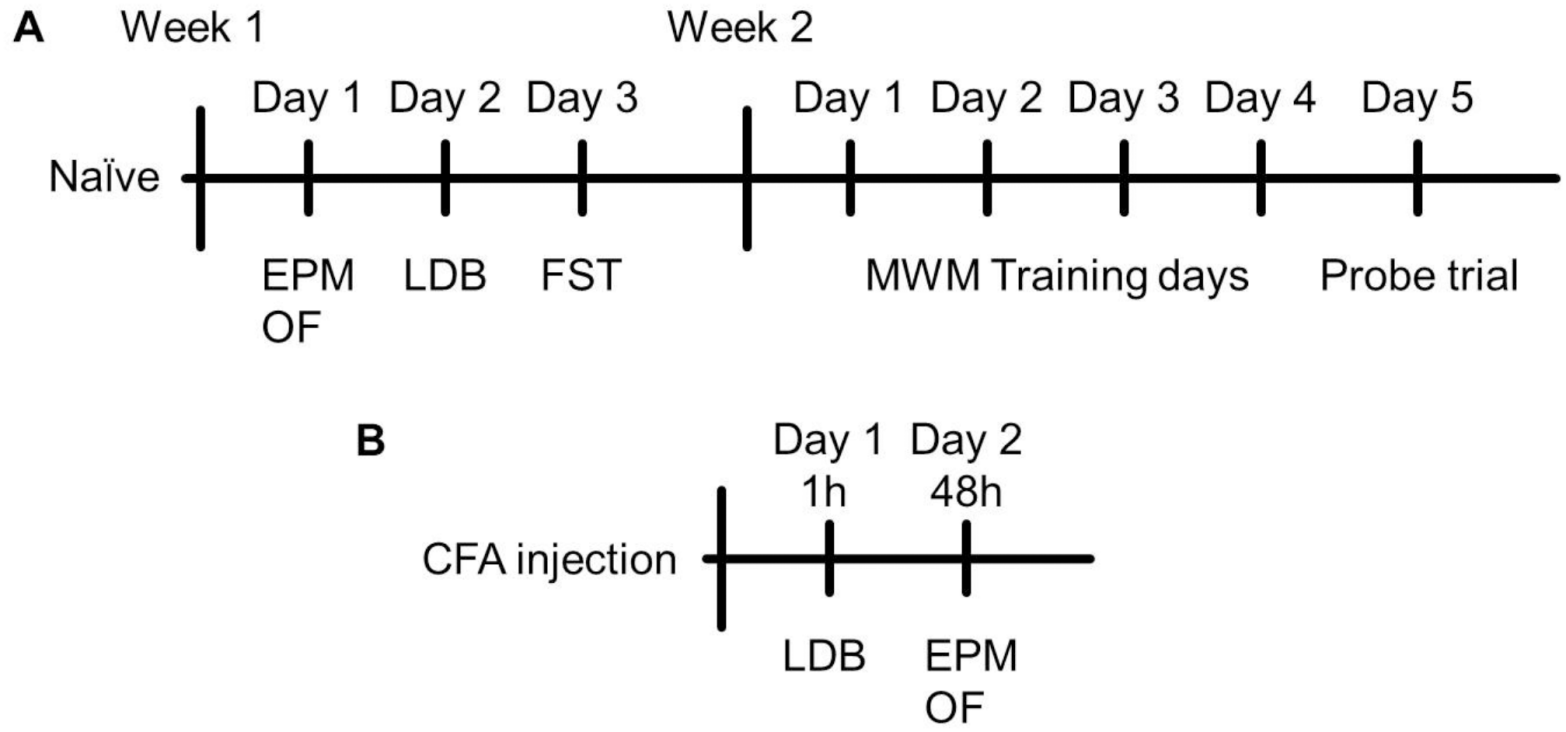
Experimental design. Timelines show the test sequences at baseline and after i.pl. injection of complete Freund`s adjuvant (CFA) into the right hind paw. Anxiety- and depression-like behavior was investigated in young (3 months) and old (≥18 months) α-GAL A deficient (Fabry KO) and wildtype littermate (WT) male mice in the naïve state on three consecutive days of the first test week and learning behavior on five days of the second test week (A). Anxiety-like behavior was additionally tested one and 48 hours after CFA injection when pain maximum was assumed [22, 23] (B). Abbreviations: EPM = elevated plus maze; FST = forced swim test; LDB = light-dark box; MWM = morris water maze; OF = open field test.

We studied 32 male Fabry KO mice carrying a targeted disruption of the α-GAL A gene as described elsewhere [11] and 32 male inbred wild type (WT) littermates of C57Bl/6N background. Fabry KO breeder pairs had been obtained with permission of Prof. A. Kulkarni (National Institute of Health, Bethesda, USA) [11]. Male Fabry KO and WT mice were separated into two age-groups of young (3 months) and old (≥18 months) animals. One cohort of both genotypes and age-groups was investigated in the naïve state and another cohort, one and 48 hours after i.pl. injection of 10 μl of complete Freund`s adjuvant (CFA, Sigma-Aldrich, Taufkirchen, Germany) into the right hind paw (concentration: 20 ng/ml, dilution: 1:100.000) under isoflurane anesthesia (CP-Pharma, Burgdorf, Germany) following the algorithm shown in Fig 1. CFA injection was used as an established model for inflammatory pain [20, 21]. All tests were video recorded for off-line analysis. Mice were housed in a reversed light-dark cycle (light cycle: 7 p.m.-7 a.m.; dark cycle: 7 a.m.-7 p.m.) and were tested during their active phase. All tests were performed by an experienced investigator blinded to the genotype (L.H.).

### Anxiety tests

The elevated plus maze (EPM) [24], the light-dark box (LDB) [25], and the open field test (OF) [26] were performed to investigate anxiety-like behavior. These three tests were chosen to cover the expected range of intra-individual outcome variation [27]. Mice were tested five minutes each. Behavioral parameters were recorded in a black box under infra-red light to avoid interference with the investigator and other mice.

The EPM apparatus consisted of two opposite open arms (66.5 cm) and two closed arms (65.5 cm), separated by a junction area. Mice were placed individually in the middle of the apparatus, facing an open arm. The total time spent in open arms was determined.

The LDB consisted of an illuminated (40 cm x 20.5 cm) and a dark compartment (40 cm x 19.5 cm), separated by an opening through which mice could switch between the compartments. Each mouse was first placed in the light box and was allowed to explore the apparatus freely. The percentage of time spent in the light box and the number of entries into the dark box were recorded.

The OF box (40 cm x 40 cm) was divided into two areas, the center zone (20 cm x 20 cm) and the surrounding area. Mice were placed individually in the middle of the center zone and the time spent in the center zone and total distance travelled was measured.

### Depression-like behavior

The forced swim test (FST) [28] was used to assess depression-like behavior of naïve young Fabry KO and WT mice. Old mice were spared due to physical exhaustion while trying to escape the stressful experimental situation. Mice were placed in a glass cylinder partially filled with water (diameter: 11.5 cm; water height: 12.5 cm; temperature: 20°C ±2°C) to observe their behavior for five minutes within a six minute period. Water level was chosen to force mice to swim or to float without touching the bottom. Time spent immobile was measured.

### Learning behavior

To test the learning behavior the Morris water maze (MWM) [29] was used. A plastic cylindrical pool (diameter: 118. 5 cm) was filled with opaque water (temperature: 20°C ±2°C) just covering the platform (diameter: 8 cm). The pool was divided into four sections and the platform was placed in the south-east (target zone) quadrant. On each of four consecutive training days mice were given four sessions of swimming with four different starting points, located in the middle of each of the four quadrants. Time spent to reach the platform was measured. If the mice were not able to find the platform within 60 seconds, they were placed on the platform for 15 seconds by the experimenter. At day five (probe trial) the platform was removed, a new starting point was selected and mice were tracked for one minute. Time spent to find the platform during the training days for each mouse was assessed and the daily average of each group was calculated. On the probe trial, time travelled in the target zone and time until first entry into the target zone was measured.

### Video processing and statistical analysis

Acquired videos were analyzed using the ANY-maze video tracking system (system version: 4.99m, Stoelting, USA). For statistical analysis SPSS IBM software Version 23 (Ehningen, Germany) and for graph design GraphPad PRISM Version 5.03 (GraphPad Software, Inc., La Jolla, CA, USA) was employed. The non-parametric Mann-Whitney U test was applied, since data were not normally distributed. Data are illustrated as box plots, representing the median value and the upper and lower 25% and 75% quartile and as bar graphs representing the mean and the standard error of the mean. Data were stratified for age groups (young: ca. 3 months, old: ≥18 months) and treatment (naïve, CFA i.pl.). P values <0.05 were considered statistically significant.

## Results

### I.pl. CFA injection induces anxiety-like behavior in the EPM in Fabry KO and WT mice

**Fig 2:**
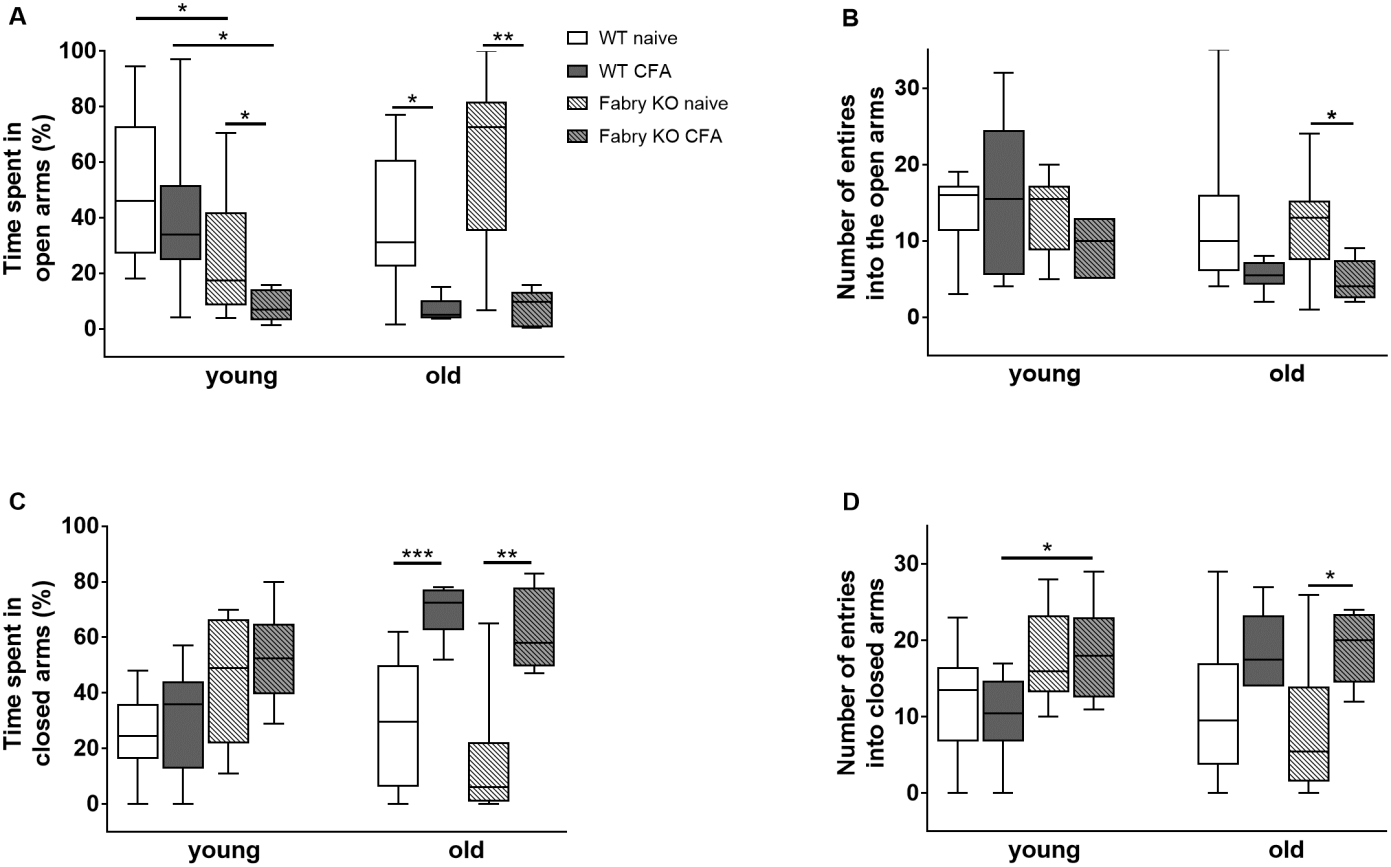
Anxiety-like behavior in the elevated plus maze test. Boxplots show the results of anxiety-like behavior in the elevated plus maze test (EPM). Young (3 months) and old (≥18 months) α-GAL A deficient (Fabry KO) and wildtype littermate (WT) male mice were investigated in the naïve state and after i.pl. complete Freund`s adjuvant (CFA) injection into the right hind paw. A) Time spent in the open arms was not different between naïve genotypes and age-groups, except for young WT mice compared to young Fabry KO mice (p<0.05). I.pl. CFA injection led to a decrease of time spent in the open arms in young Fabry KO mice compared to young naïve Fabry KO mice (p<0.05). After CFA injection time spent in open arms was also lower in young Fabry KO mice than in young WT mice (p<0.05). In old mice CFA i.pl. reduced time spent in open arms in Fabry KO (p<0.01) and WT mice (p<0.05). B) There was no intergroup difference in the number of entries into the open arms in naïve and CFA treated mice except for old CFA treated Fabry KO mice, which entered less often than old naïve Fabry KO mice (p<0.05). C) No difference was found in young age-groups for time spent in closed arms. Old CFA treated mice of both genotypes spent more time in the closed arms compared to old naive mice (Fabry KO: p<0.01; WT: p<0.001). D). There was no difference in entries into the closed arms between age-groups and genotypes in the naïve state. After CFA injection young Fabry KO mice more often entered the closed arms than young WT mice (p<0.05). Also, old CFA treated Fabry KO mice had more entries into the closed arm than old naïve mice (p<0.05). Fabry KO: young (3 months; naïve: 10 male; CFA: 6 male), old (≥18 months; naïve: 10 male; CFA: 6 male). WT: young (3 months; naïve: 10 male; CFA: 6 male), old (≥18 months; naïve: 10 male; CFA: 6 male). *p<0.05, **p<0.01.

In the EPM no genotype- or age-associated differences were found for time spent in the open arms and entries into the open arms when comparing naïve mice (Fig 2A and 2B), except for longer time spent in open arms by young WT mice compared to young Fabry KO mice (p<0.05; Fig 2A). I.pl. CFA injection reduced time spent in the open arms compared to baseline in young (Fabry KO: p<0.05; WT: n.s.; Fig 2A) and old mice (Fabry KO: p<0.01; WT: p<0.05; Fig 2A). The reduction was particularly visible in young Fabry KO mice compared to young WT mice (p<0.05; Fig 2A). CFA injection also reduced the number of entries into the open arms in old Fabry KO mice (p<0.05; Fig 2B).

Young and old age-groups showed no intergroup difference in time spent in closed arms (Fig 2C). I.pl. CFA injection induced an increase in time spent in closed arms only in old mice of both genotypes (Fabry KO: p<0.01; WT: p<0.001; Fig 2C). CFA injection also increased the number of entries into the closed arms in young Fabry KO mice compared to young treated WT mice (p<0.05) and old Fabry KO mice compared to baseline (p<0.05; Fig 2D).

**Fig 3:**
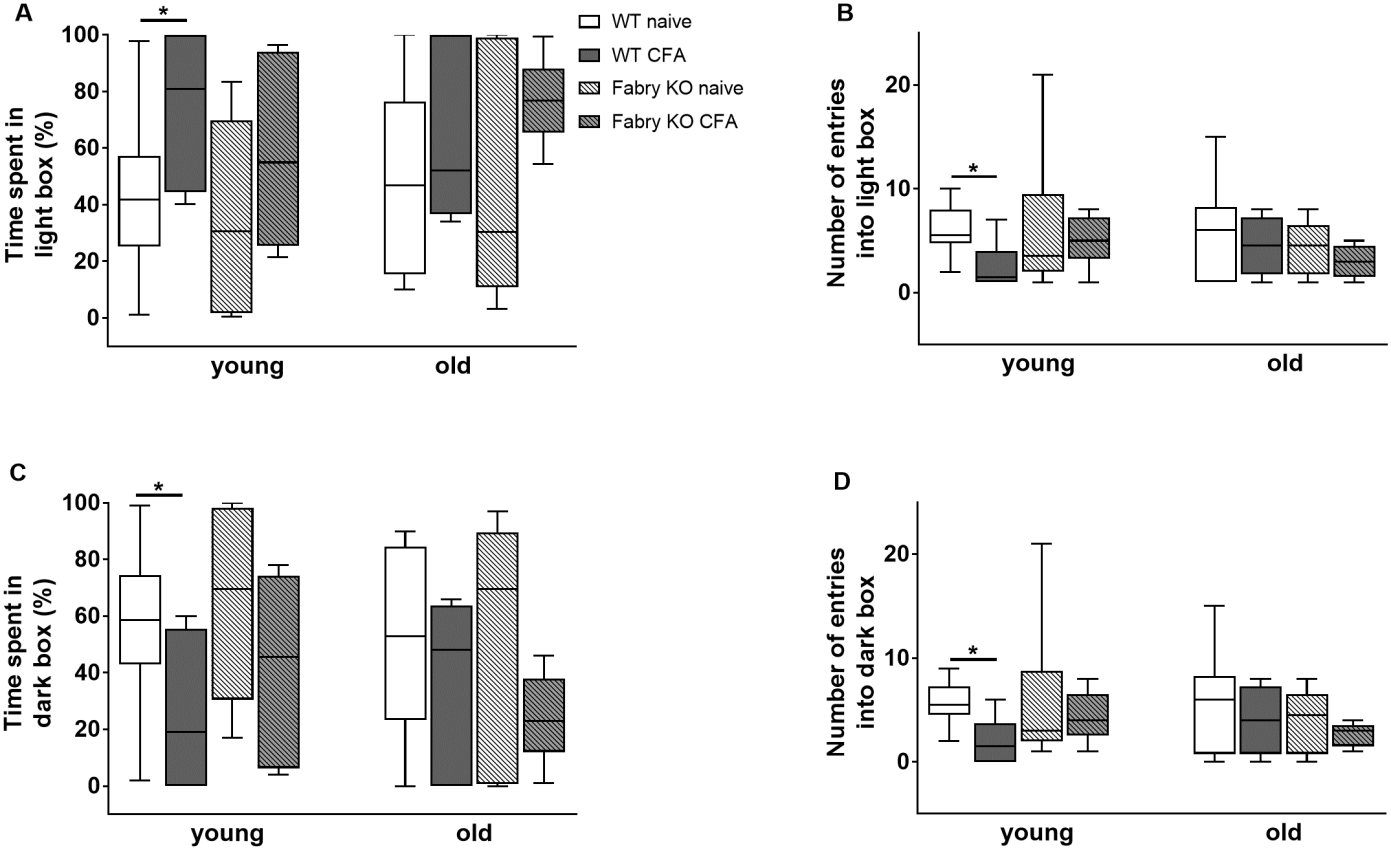
Anxiety-like behavior in the light-dark box test. Boxplots show the results of anxiety-like behavior in the light-dark box test (LDB). Young (3 months) and old (≥18 months) old α-GAL A deficient (Fabry KO) and wildtype littermate (WT) male mice were investigated in the naïve state and after i.pl. complete Freund`s adjuvant (CFA) injection into the right hind paw. There was no intergroup difference between genotypes, age-groups, and treatment for the time spent in the light box (A), entries into the light box (B), time spent in the dark box (C) and entries into the dark box (D) except for the comparison of young naïve WT mice with young CFA treated WT mice (p<0.05 each). Fabry KO: young (3 months; naïve: 10 male; CFA: 6 male), old (≥18 months; naïve: 10 male; CFA: 6 male). WT: young (3 months; naïve: 10 male; CFA: 6 male), old (≥18 months; naïve: 10 male; CFA: 6 male).

**Fig 4:**
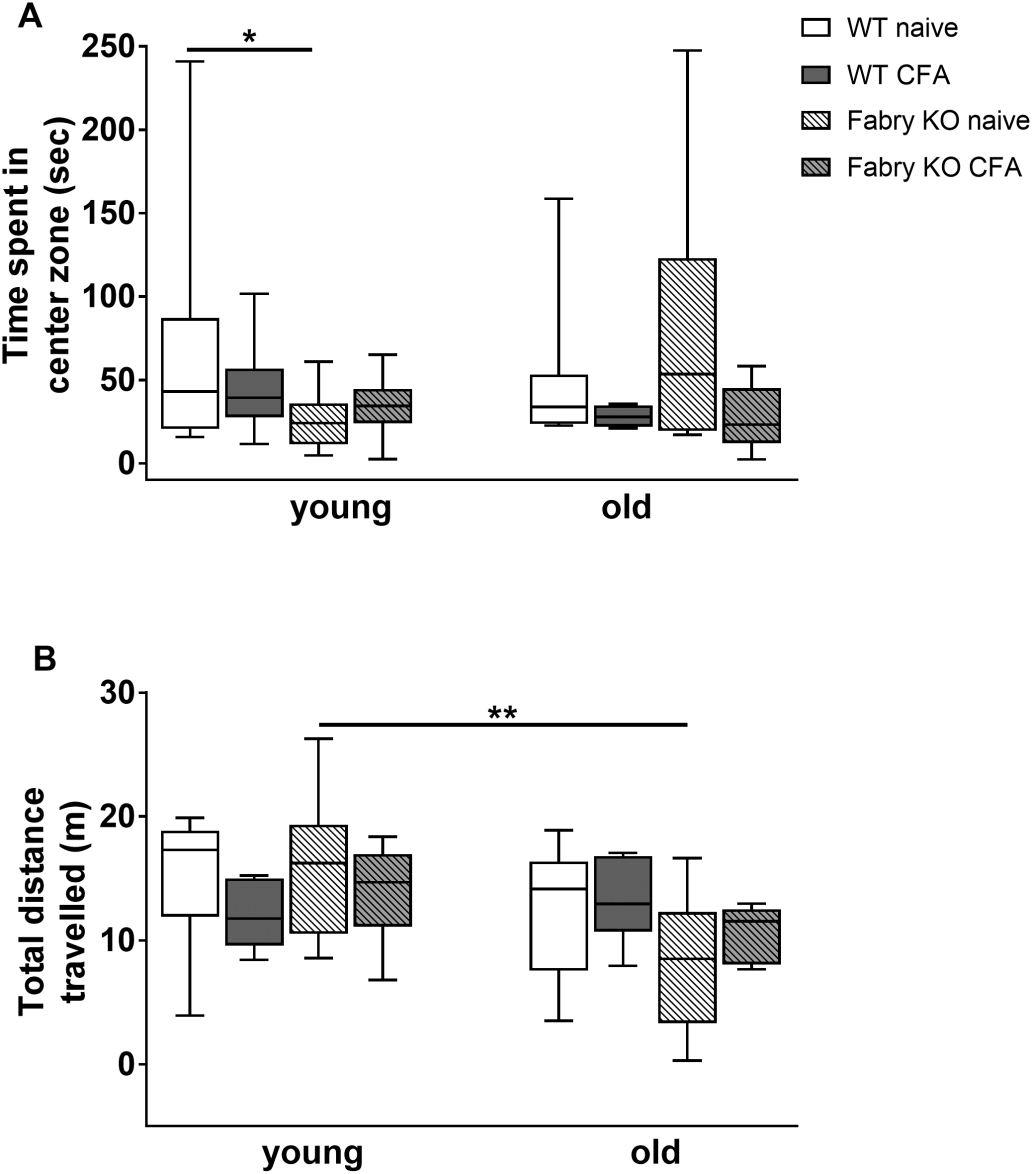
Anxiety-like behavior in the open field test. Boxplots show the results of the anxiety-like behavior in the open field test (OF). Young (3 months) and old (≥18 months) old α-GAL A deficient (Fabry KO) and wildtype littermate (WT) mice were investigated in the naïve state and after i.pl. complete Freund`s adjuvant (CFA) injection into the right hind paw. No difference between genotypes, age- and treatment groups was found in the time spent in the center zone (A), except for young WT mice compared to young Fabry KO mice in the naïve state (p<0.05). Total distance travelled (B) was not different between genotypes, age- and treatment groups except for young Fabry KO mice in the naïve state compared with old Fabry KO mice (p<0.01). Fabry KO: young (3 months; naïve: 10 male; CFA: 6 male), old (≥18 months; naïve: 10 male, CFA: 6 male). WT: young (3 months; naïve: 10 male; CFA: 6 male), old (≥18 months;naïve: 10 male; CFA: 6 male). *p<0.05, **p<0.01.

In the LDB experiment time spent in the light and dark box and the number of entries into the light and dark box did not differ between genotypes, age- and treatment groups, except for young WT mice in the naïve state compared to CFA treated young WT mice (p<0.05 each; Fig 3). Time spent in the central central zone of the OF only differed between young WT and young Fabry KO mice in the naïve state (p<0.05; Fig 4A). Total distances travelled in the OF were longer for naïve young Fabry KO mice compared to old Fabry KO mice (p<0.01; Fig 4B).

### No depression-like behavior in Fabry KO mice

**Fig 5:**
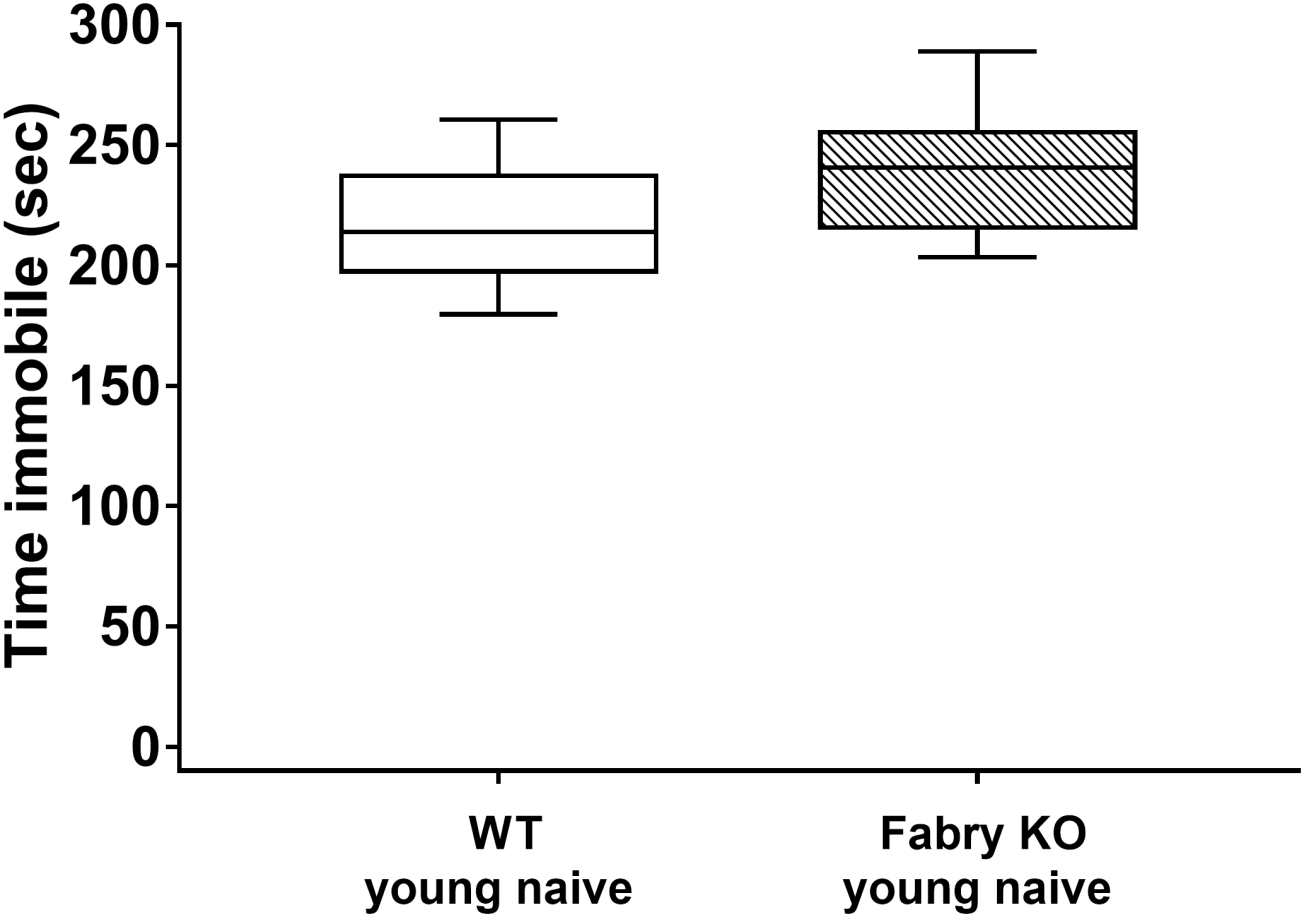
Depression-like behavior in the forced swim test. Boxplots show the results of the depression-like behavior in the forced swim test (FST). Young naive (3 month) α-GAL A deficient (Fabry KO) and wildtype (WT) male mice were investigated. No difference was found between the genotypes. Fabry KO: young (3 months; 10 male). WT: young (3 months; 10 male).

The assessment of potential depression-like behavior in the FST revealed no genotype differences. Naïve Fabry KO and WT mice spent similar times floating in the water basin (Fabry KO: median 180.8 sec, range 160.6–221.4 sec; WT: median 154.6 sec, range 127.6–225.7 sec; Fig 5).

### Similar learning behavior in Fabry KO and WT mice

**Fig 6:**
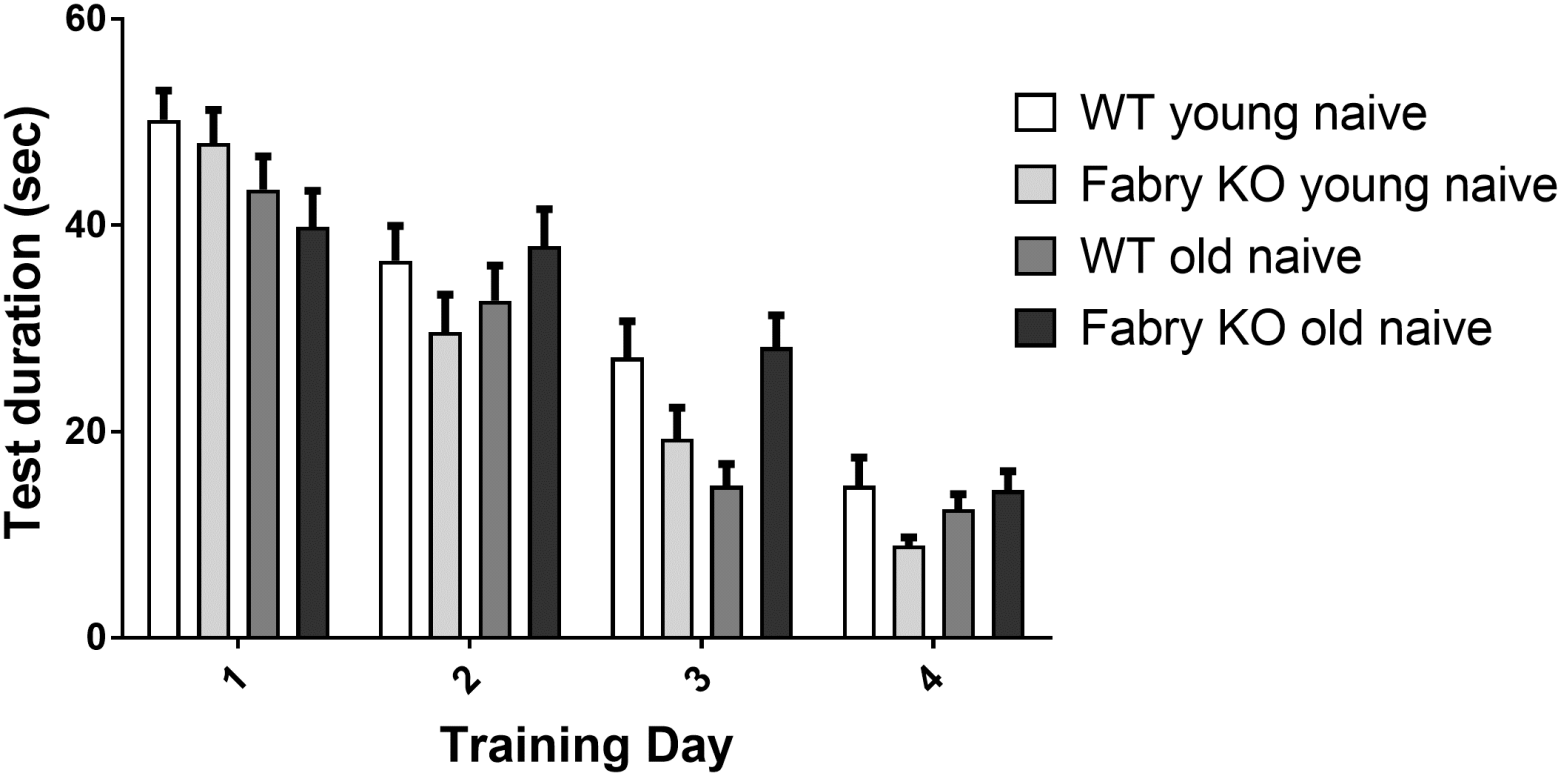
Learning behavior in the Morris water maze test. Bar graphs show the results of the learning behavior in the Morris water maze test (MWM). Naive young (3 months) and old (≥18 months) α-GAL deficient (Fabry KO) and wildtype littermate (WT) male mice were investigated. Test duration on training days displayed not relevant differences between genotypes and age-groups and continuously decreased from training day one to four. Fabry KO: young (3 months; 10 male), old (≥18 months; 10 male). WT: young (3 months; 10 male), old (≥18 months; 10 male). *p<0.05, **p<0.01.

**Fig 7:**
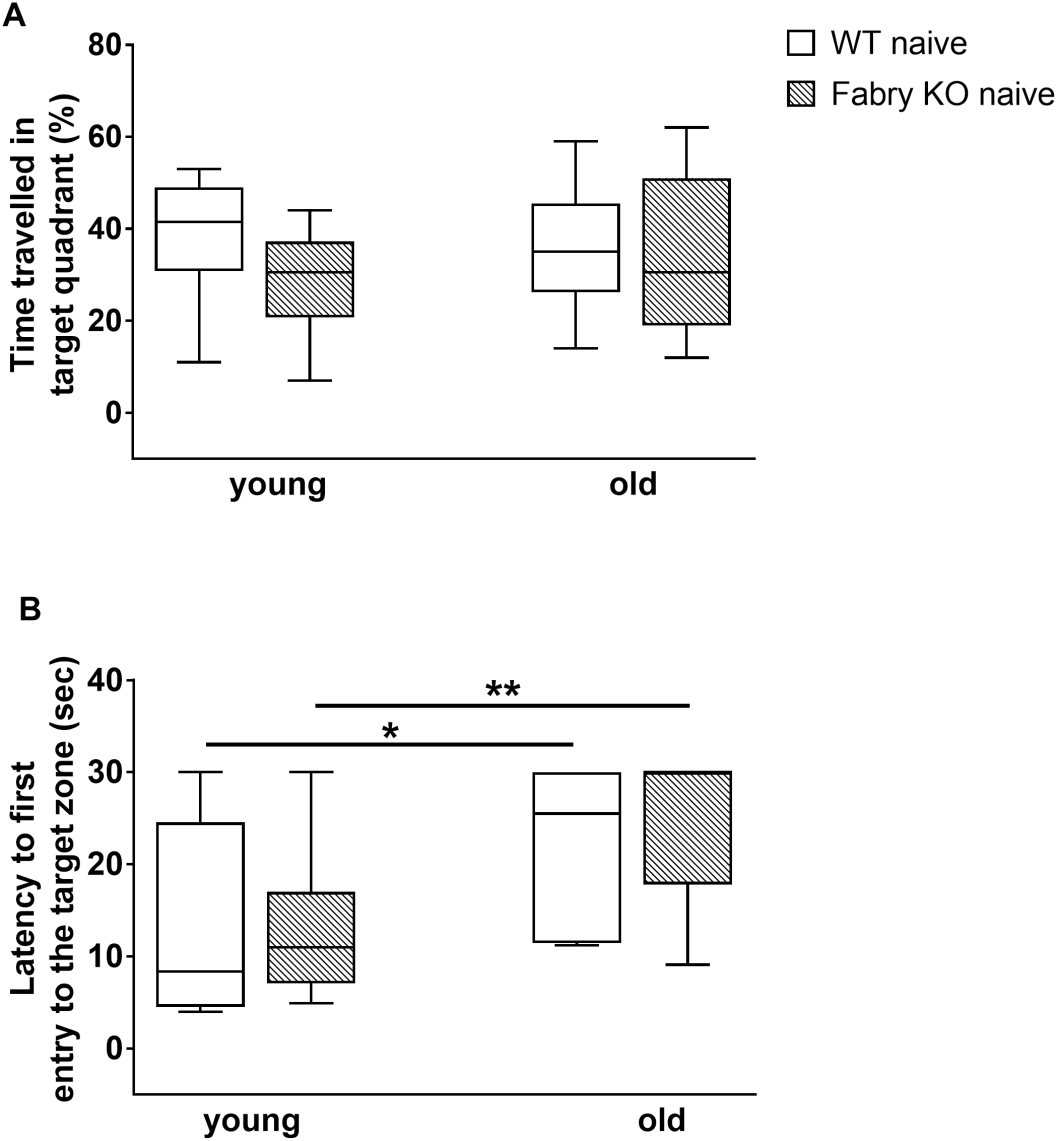
Memory function in the Morris water maze test. Boxplots show the results of the memory function in the Morris water maze test (MWM). Naive young (3 months) and old (≥18 months) old α-GAL A deficient (Fabry KO) and wildtype littermate (WT) male mice were investigated. Time travelled in target quadrant (A) did not differ between genotypes and age-groups. Latency until first entry into the target zone (B) was shorter for mice of the young age-group compared to old mice (Fabry KO: p<0.01; WT: p<0.05). Fabry KO: young (3 months; 10 male), old (≥18 months; 10 male). WT: young (3 months; 10 male), old (≥18 months; 10 male). *p<0.05, **p<0.01.

In the training session no relevant differences were found in mice of both genotypes and age-groups for total test duration; time spent until finding the hidden platform continuously decreased from training day one to four (Fig 6). In the probe trial, time travelled in the target quadrant was not different between genotypes and age-groups (Fig 7A). As expected, the latency until first entry into the target zone was shorter for young compared to old mice of both genotypes (Fabry KO: p<0.01; WT: p<0.05; Fig 7B).

## Discussion

We characterized affective and learning behavior of α-GAL A deficient mice [11] asking whether there might be neuropsychological symptoms, as frequently reported by FD patients. The presence of such findings in the mouse model would favor a genetic cause, while, alternatively, these symptoms might be regarded as a sequel of environmental influences, insufficient coping strategies, or other factors secondary to this often painful and life-threatening disease. Since we did not find major differences between Fabry KO and WT mice in our study, we cannot support the notion of a major genetic influence on neuropsychological symptoms.

Anxiety is a frequent psychological symptom reported by FD patients [7, 9, 30, 31]. Prevalence estimates in current literature range between 20 and 37% [31, 32]. Since the intra-individual outcome variation is particularly high in anxiety tests for animals [27], we performed three different assays before and after CFA treatment [20, 21]. The comparison of genotype- and age-groups in the naïve state did not reveal relevant differences. CFA injection resulted in an increase in anxiety-like behavior particularly in Fabry KO mice, however, this was only reflected in the EPM (Fig 1). In contrast, Fabry KO and WT mice displayed similar anxiety-like behavior patterns in the LDB and the OF at baseline and after CFA treatment. One reason might be that all three tests are exploration-based mouse tasks. Thus, diverse results in EPM, LDB, and OF for anxiety-like behavior are plausible due to the diversity in environmental settings of the assays used (e.g. illuminated box in the LDB versus no light stimulus in the EPM test). This diversity rather underlines the necessity to apply multiple tests at different time points, because each mouse can display differences in emotional behavior during the test phase. Therefore it is essential to interpret results after amalgamation of the obtained spectrum of data [27, 33, 34]. It is also of note that we used a rather low CFA concentration (20 ng/ ml/injection) not to miss potential mild intergroup differences. Using a higher CFA concentration (e.g. 1 mg/ml/injection) as reported in previous studies [19] may have had resulted in consistent anxiety-like behavior in more than one test paradigm.

While our data do not support a genetic basis of affective and cognitive alterations in FD patients, the presence of chronic pain may be one contributor. The majority of men and women with FD do suffer from episodic and/or permanent pain [2, 35, 36] mostly starting in early childhood and limiting patients`physical activities, everyday life, and working and learning capacities [3, 5, 7]. This may already induce depressive symptoms and also impair cognitive functions such as concentration and mental endurance as known e.g. for patients with fibromyalgia syndrome [37] or other neuropathic pain syndromes like diabetic peripheral neuropathy or complex regional pain syndrome [38]. The underlying pathomechanisms linking pain, affective, and cognitive behavior are not fully understood, however, there are several potential factors that might play a pathophysiological role. Pro- and anti-inflammatory cytokines, for instance, are involved in the pathophysiology of chronic pain [39] and modulate hippocampal neurogenesis altering the emotional state of the patients [40, 41]. In peripheral blood mononuclear cells of FD patients, a pro-inflammatory cytokine expression profile was reported including elevated levels of interleukin 6 (IL-6) and IL-1β [42], which may both decrease neuronal serotonin levels resulting in depressive symptoms [43]. Additionally, it has been shown that physiological levels of IL-1β play a role in the maintenance of long term potentiation (LTP) [44], which is important for hippocampus-dependent learning and cognition. In contrast to this, increased IL-1β levels in the hippocampus during pathological and inflammatory states are associated with inhibition of LTP induction which may impair cognitive and memory functions [45].

Most studies in FD patients focus on depression [7, 10] and cognitive functions [4, 6, 7, 46] applying questionnaires and computerized assessment systems. The overall finding is that depressive symptoms are frequent in FD patients reaching up to 60% of the investigated study cohort [5]. Our experiments gave no hints for increased depression-like behavior in α-GAL A deficient mice which argues against a genetically determined depressive mood in FD patients. However, in addition to the limitations when comparing mouse and man, our data need to be interpreted with caution since mice investigated in this study were originally created by targeted disruption of the α-GAL A gene in mouse embryonic cells, thus no α-GAL A activity is present [11]. This is in contrast to the situation in human patients, where enzyme activity can range between normal and complete loss [1] and clinical phenotype is substantially determined by the underlying distinct genetic alteration [47–49].

The assessment of cognitive function in FD patients so far mostly revealed mild and unprogressive symptoms [4, 6, 7]. The lack of genotype differences in learning behavior of our FD mouse model is therefore in line with findings in Fabry patients. However, evidence reflecting the characteristic cognitive phenotype observed in FD patients is limited [5], which hampers adequate data interpretation. Also, appropriate methods and tools to more effectively investigate and comprehend the distinct Fabry-associated psychological phenotype are still missing.

Despite these limitations, our study provides important insights into affective and cognitive behavior in α-GAL A mice. We applied a comprehensive battery of behavioral tests and compared Fabry KO and WT littermate mice at young and old age, which is essential because age-dependent behavioral changes are known in α-GAL A deficient mice [14].

## Conclusions

Affective and learning behavior seem to be spared from the Fabry genotype. In contrast to a previous study [19], we found no influence of CFA-induced inflammatory pain on anxiety behavior. Extrapolating to FD patients, the genetic influence on affective and cognitive symptoms in FD patients may be of subordinate importance. Further studies are needed to clarify the potential relevance of these findings in human patients.
